# Incidental Myocardial Infarction on Routine Non-Gated Thoracic Computed Tomography

**DOI:** 10.2174/0115734056363863250509093505

**Published:** 2025-05-19

**Authors:** Mehrad Rokni, Yasser G. Abdelhafez, Lorenzo Nardo, Mohammad H. Madani

**Affiliations:** 1 Department of Radiology, University of California, Davis Medical Center, 4860 Y St, Suite 3100, Sacramento, CA 95817, United States of America

**Keywords:** Thoracic Computed Tomography, Incidental Myocardial Infarction, Major Adverse Cardiovascular Events, Survival, Coronary artery calcifications, Percutaneous coronary intervention

## Abstract

**Aims:**

The aim of this study is to assess the identification of incidental myocardial infarction on non-electrocardiogram-gated computed tomographic scans of the chest and its prognostic significance.

**Background:**

The increased utilization and abundance of thoracic computed tomographic (CT) scans have provided a substrate for potential screening purposes.

**Objective:**

The objective of this study was to evaluate the detection of incidental myocardial infarction on routine non-gated thoracic CT performed for non-cardiac reasons and its associated major cardiovascular events and survival.

**Methods:**

We retrospectively assessed routine non-gated thoracic CT scans of all consecutive individuals aged 18 or above who underwent thoracic CT scans as outpatients at the University of California Davis from January 2015 to December 2015. We evaluated the presence and location of incidental MI on non-gated thoracic CT and compared major adverse cardiac events (MACE) and overall survival in CT-positive infarct individuals with a CT-negative infarct control group.

**Results:**

We reviewed routine thoracic CT scans of 1157 individuals and identified 12 individuals with incidental MI. The mean age of individuals with infarction was 71.4 ± 14.1 years, and 50% were female. All individuals with incidental MI had coronary calcification. Individuals with incidental MI had a higher rate of MACE endpoint (92% *vs*. 28%, p=0.0001), number of MACE events (1.1 *vs*. 0.3, p<0.001), and lower overall survival (median survival of 67 months *vs*. not reached, p=0.023) compared with age and sex-matched controls without incidental MI.

**Conclusion:**

Although small in number relative to the total number of individuals evaluated, subjects with incidental MI on routine non-gated thoracic CT scans have worse cardiovascular outcomes and survival compared with controls without infarction. This study highlights the potential opportunistic screening utility of routine thoracic CTs, which could lead to improved risk stratification and intervention.

## 1 INTRODUCTION

The utilization of medical imaging has grown over the past two decades. Roughly 85 million thoracic CTs are now performed annually in the US [1]. The plethora of imaging provides opportunities for screening purposes unrelated to the primary imaging indication. Myocardial infarction is a leading cause of mortality and morbidity; however, it may not be recognized by clinical exam or electrocardiogram. There are case reports and series that have documented the phenomenon of incidental myocardial infarction (MI) in CT studies [2, 3]. In addition to these reports and series, there have been a small number of prior systematic studies on cross-sectional imaging detection of incidental myocardial scar or hypoattenuation, such as studies by Kuetting *et al*. [4], Sverzellati *et al*. [5], and Turkbey *et al*. [6] Prior systematic studies have assessed myocardial fat, not specifically infarct, selected particular patient populations, or employed magnetic resonance imaging (MRI) instead of CT.

This study aims to investigate the detection of incidental myocardial infarction on routine thoracic CT scans as well as the prognostic implications of such detection by examining the occurrence of major adverse cardiovascular events and overall survival.

## 2 MATERIALS AND METHODS

The study is a retrospective, cross-sectional analysis of all consecutive adults 18 years or older who underwent routine outpatient non-gated thoracic CT at the University of California, Davis in Sacramento, California, between January 2015 and December 2015 (n=1157 subjects). This study was performed in line with the principles of the Declaration of Helsinki. Approval was granted by the University of California Davis Institutional Review Board (IRB ID: 2040249-1). The requirement for informed consent was waived due to the retrospective nature of the study.

### 2.1 CT Protocol, Indications, and Data Collection

CT scans were performed using a standard non-gated contrast thoracic CT protocol on a 64-slice multidetector CT scanner. The scanning parameters were as follows: tube voltage of 120 kVp, automatic tube current modulation, slice thickness of 1 mm, and reconstruction interval of 0.8 mm.

There were a variety of indications of the non-gated thoracic CT scans (Table **1**). The most frequent indication of a CT scan was oncologic staging and follow-up (50%) and evaluation of solitary pulmonary nodules (15%). Other indications included dyspnea and respiratory failure (6%), chest radiographic abnormality (6%), anatomic evaluation (5%), evaluation for thromboembolism (5%), cough (3%), infection (2%), and lung cancer screening (2%).

### 2.2 Coronary Artery Calcifications

Coronary artery calcifications were evaluated using Hounsfield unit thresholds and categorized into mild (1-100), moderate (101-400), or severe (>400) based on their density and extent. The presence and severity of coronary artery calcifications were documented for each patient.

### 2.3 MACE Calculation

MACE was determined as a composite cardiovascular endpoint, including cardiovascular death, non-fatal myocardial infarction, non-fatal stroke, and coronary revascularization procedures such as percutaneous coronary intervention (PCI) or coronary artery bypass grafting (CABG). The occurrence of MACE events was tracked through medical records and follow-up assessments, ensuring comprehensive outcome evaluation.

We identified 12 patients who were positive for infarction, as described below in the image analysis section. These patients were then matched with 36 patients who were negative for infarction, forming a control group. The matching was done based on age and sex to ensure comparability between the groups. Clinical characteristics data for each patient, cardiovascular risk factors, and all mortality causes were gathered. Major Adverse Cardiovascular Events (MACE) were calculated, and the patients were followed up until October 2023.

### 2.4 Image Analysis

CT images of patients were initially screened for myocardial infarction by a physician-scientist with 9 years of clinical experience and who was blinded to the patients’ clinical details. Myocardial infarct in our study was defined as myocardial hypoattenuation with subendocardial involvement, corresponding with coronary artery distribution and not due to artifacts. Potential cases of myocardial infarction were then reviewed and confirmed by a cardiothoracic radiologist with 10 years of clinical experience, also blinded to patients’ clinical information. Location of myocardial infarct based on American Heart Association segmentation [7], transmural extent of the infarct, myocardial density, and other associated cardiovascular findings, including presence/degree of coronary artery calcifications in patients with myocardial infarct, were also recorded.

### 2.5 Statistical Analysis

In this study, data were summarized as counts and percentages for categorical variables or as the mean and standard deviation (SD) for continuous variables. A control group was selected using a propensity score matching approach, according to sex, age, and BMI, in a ratio of 3-to-1 (negative-to-positive ratio).

The association of independent categorical characteristics with the CT finding of myocardial infarction (two groups) was examined using Fisher’s exact test. Since continuous variables of interest were not normally distributed, the Mann-Whitney U test was used to compare the median ranks of continuous data among the two groups (with positive and negative CT findings). Time-to-event was calculated from the date of the reference CT study to the date of death from any cause or date of censoring at their last follow-up.

For survival analysis, only the patients who completed at least 6 months before censoring or death were included. Survival data were analyzed using the Kaplan-Meier method, and the log-rank test was used to compare the overall survival probability between patients with and without CT evidence of infarction. All analyses were performed using SPSS v.29 (IBM, Armond, New York, USA). To assess the study’s ability to detect observed differences, a post-hoc power analysis was conducted using G*Power software (version 3.1) with α = 0.05 (two-tailed).

## 3 RESULTS

### 3.1 Patient Characteristics

In our retrospective study, we analyzed the clinical characteristics of subjects at baseline across two groups: CT infarct positive subjects and CT infarct negative controls. The combined groups had an average age of 71.2 ± 13.3 years and a BMI of 25.6 ± 6.3 kg/m^2^. Approximately half were female, and the majority of the population was white (70%). Most patients had hypertension (69%) and hyperlipidemia (71%), while a smaller percentage had diabetes (19%). Forty percent had a history of coronary artery disease, and 69% had a smoking history.

There was no significant difference between the positive and negative infarct groups in age, BMI, sex, and race/ethnicity, which had mean ages of 71.4 ± 14.1 years and 71.1 ± 13.3 years, respectively. The positive (infarct) group and the negative (non-infarct) group show significant differences in hypertension, diabetes, coronary artery disease (CAD), and smoking status. All individuals in the positive group have hypertension, compared to 58% in the negative group, a statistically significant difference (p=0.009). Diabetes is present in 50% of the positive group, significantly higher than the 8% in the negative group (p=0.004). A large majority of the positive group have CAD (83%), significantly more than the 25% in the negative group (p=0.0006). All individuals in the positive group are smokers, compared to 40% in the negative group, a statistically significant difference (p=0.029). The corresponding data for these indications is summarized in Table **2**. All infarct patients were asymptomatic for cardiac symptoms at the time of CT, including no active chest pain or clinical signs of ischemia.

### 3.2 Imaging Features of the Infarct Group

Table **3** delineates the imaging characteristics of myocardial infarcts, with a particular emphasis on the frequency of hypoattenuation across distinct myocardial segments and the transmurality of the infarcted regions. American Heart Association segments 2, 4, 5, 9, and 17 manifested a frequency of 4.4%, while segment 7 demonstrated the highest frequency at 15.5%. In terms of the transmurality of the infarcted regions, 25% of the infarcts exceeded 50% of the myocardial thickness, 50% were within the range of 25% to 50% of the myocardial thickness, and the remaining 25% were less than 25% of the myocardial thickness. Representative CT images of incidental left ventricular myocardial infarctions are shown in Figs. (**1** and **2**).

### 3.3 Coronary Artery Calcifications Association

We found a significant association between coronary calcifications and the results. All patients with CT positive for infarction, representing 100% of this group, exhibited coronary calcifications, compared to 67% in the CT infarct negative group (p=0.023). Severe calcifications were universally present in patients who manifested myocardial infarction during the follow-up period. Additionally, moderate calcifications were statistically significantly associated with the CT-positive infarct group (p=0.011). Interestingly, the mean Hounsfield units of normal myocardium were relatively similar between both groups, with values at 85.2±21.5 for the CT infarct negative group and 82±23 for the CT positive infarct group, yielding a non-significant p-value of 0.7. These findings are illustrated in Table **4**.

### 3.4 MACE Outcomes and Survival

The Major Adverse Cardiac Events (MACE) endpoint demonstrated a significant association with the results. Among patients with a CT positive for infarction, 92% (6 MIs, 2 MI and then coronary artery bypass graft (CABG), 2 strokes, and just 1 CABG alone) experienced a MACE endpoint—a composite of cardiovascular death, non-fatal myocardial infarction, and non-fatal stroke—compared to 28% (3 MIs, 1 MI and then CABG, 3 strokes, 2 transient ischemic attack (TIA) and just 1 CABG alone) in the CT negative for infarct group (p=0.0001). Furthermore, the average number of MACE events was significantly higher in patients with a positive CT infarct result (1.1±0.5) than in those with a negative CT result (0.3±0.5) (p<0.001), as indicated in Table **5**.

**Fig. (3):**
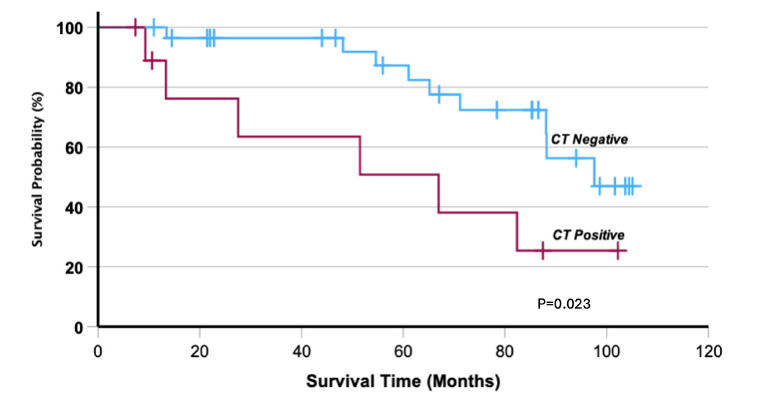
Survival Rates in CT Positive versus CT Negative Myocardial Infarct Individuals. Kaplan-Meier survival curve comparing survival rates between CT positive and CT negative myocardial infarct individuals. The curve highlights the significant difference in survival outcomes between the two groups.

The median follow-up for the whole group was 53.1 months. Nine patients did not complete the minimum 6 months, leaving a valid cohort of 39 patients. Six out of the nine patients were lost to follow-up due to cancer-related deaths, such as bladder cancer, lung cancer, pancreatic cancer, mediastinal adenocarcinoma, and renal cell carcinoma. The remaining three were lost to follow-up. Overall survival was significantly lower in the CT positive infarct group, 6 events out of 10 and the median survival time was 67.0 months (14.6-119.4), compared to 9 out of 29 patients in the CT negative infarct group (Fig. **3**, P = 0.023).

For the MACE endpoint (92% *vs*. 28%), power exceeded 95% (Cohen’s *h* ≈ 1.44), and for the number of MACE events (1.1 ± 0.5 *vs*. 0.3 ± 0.5), power was also >95% (Cohen’s *d* ≈ 1.6), reflecting large effect sizes. For overall survival (6/10 *vs*. 9/29 events), power was 58% (estimated HR ≈ 1.94, 15 events), limited by the smaller effective sample size and fewer events after exclusions.

## 4 DISCUSSION

Our study stands out from previous literature due to its systematic approach and evaluation of the prognostic significance of incidental myocardial infarct detection. Prior systematic studies have been performed that evaluated for myocardial fat (not specifically infarct) or did not examine the prognostic importance of myocardial hypoattenuation. A study by Turkbey *et al*. examined the prevalence of myocardial scars in middle and older-aged individuals and utilized MRI [6], which is superior to routine CT for myocardial tissue characterization. However, in many instances, patients referred for chest imaging only undergo CT instead of MRI for various non-cardiac related reasons. Kuetting *et al*. reported incidental cardiac findings, including myocardial hypoperfusion on non-gated thoracic CT scans however, all patients who underwent CT were in the intensive care unit. Sverzellati *et al*. [4] included only patients with pulmonary disease more likely to be associated with cardiovascular disease and assessed for fatty attenuation left ventricular wall [5]. Our study did not select patients who are more likely to have cardiovascular disease, further broadening the scope of our research and specifically evaluating for infarct.

Our findings demonstrate a strong association between incidental myocardial infarction, coronary artery calcification (CAC), and adverse cardiovascular outcomes, reinforcing the prognostic significance of CAC burden in cardiovascular risk assessment. All infarct-positive patients exhibited CAC, and severe calcification was associated with higher MACE incidence, aligning with prior studies such as the Multi-Ethnic Study of Atherosclerosis (MESA) [8] and the work of Wetscherek *et al*. [9], which identified CAC as a powerful predictor of cardiovascular events and mortality. Additionally, Sverzellati *et al*. [5] and Yacoub *et al*. [10] highlighted the frequent underreporting of cardiovascular findings on routine CT, leading to missed opportunities for early intervention. Given that CAC scoring is well-established in dedicated cardiac CT protocols but often overlooked in non-gated thoracic CT scans, our study underscores the potential role of routine thoracic CT as an opportunistic screening tool for cardiovascular risk stratification. The high MACE endpoint rate (92%) in infarct-positive patients suggests that incidental MI and CAC findings on routine CT scans should not be disregarded, warranting further investigation and structured reporting to enhance patient risk assessment and clinical management.

There was an extended follow-up period in our study which was a median of 53.1 months. Although the number of individuals found to have incidental myocardial infarction existed small compared with the overall number of individuals evaluated, we observed that the presence of incidental MI correlated with a higher MACE endpoint rate (92% *vs*. 28%, p=0.0001), increased MACE events (1.1 *vs*. 0.3, p<0.001), and decreased overall survival in positive infarct individuals compared with age and sex-matched controls without infarct (P = 0.023), emphasizing the prognostic value of our results. The results indicate robust power for MACE outcomes but suggest a cautious interpretation of survival findings due to sample size constraints. Our findings align with those of Wetscherek *et al*. [9] who focused on the correlation between coronary artery calcification on non-contrast, non-ECG-gated CT thorax and the risk of subsequent cardiovascular disease events. Our study featured specifically infarct detection on non-gated routine thoracic CT scans, which could be utilized for potential cardiac data extraction.

Our study demonstrated that patients with incidental MI on non-gated routine thoracic CT scans experienced worse clinical outcomes compared with controls lacking CT evidence of myocardial infarction. The large volume of routine thoracic CT scans conducted for reasons unrelated to cardiac issues offers a rich resource for potential opportunistic screening [11]. This could allow for enhanced risk stratification of patients and management.

This study has inherent limitations, including its retrospective design, which limits causal inferences and may introduce selection bias. The small sample size, particularly the number of patients with incidental myocardial infarction, affects the statistical power and generalizability of the findings. Infarct detection on non-gated thoracic CT scans is subject to image quality and reader interpretation, potentially contributing to variability. Although propensity score matching was applied to account for confounding factors, residual confounding due to unmeasured variables cannot be excluded. Future prospective studies with larger cohorts and standardized imaging protocols are needed to further validate these findings and assess their broader clinical implications.

## CONCLUSION

Our research demonstrated that incidental myocardial infarction can be detected on non-gated thoracic CT scans. The identification of these infarcts on routine imaging underscores the potential for opportunistic screening in individuals who may not otherwise undergo dedicated cardiac imaging. Given the significant association between incidental myocardial infarction and adverse cardiovascular outcomes, our findings highlight the importance of recognizing these findings as potential markers for increased cardiovascular risk. Early identification through thoracic CT could prompt further diagnostic evaluation and clinical management, leading to improved patient outcomes. Future prospective research should focus on larger-scale studies to validate these findings, assess the clinical impact of early detection, and explore potential strategies for integrating incidental myocardial infarction detection into routine radiology reporting.

Although small in number compared with the total number of subjects evaluated, individuals found to have incidental myocardial infarction on routine thoracic CT experienced greater adverse clinical outcomes compared with controls lacking CT evidence of myocardial infarction, including greater major cardiovascular events and poorer overall survival. This underscores the prognostic relevance and opportunistic screening for incidental MI through routine scans.

## Figures and Tables

**Fig. (1):**
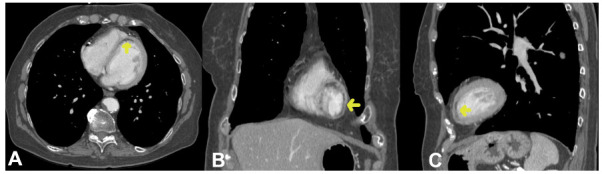
Left Ventricular Myocardial Infarction Detected on Routine Non-Gated Thoracic CT. Incidental left ventricular myocardial infarction identified in an individual who underwent a routine thoracic CT scan for the follow-up of a left lower lobe pulmonary nodule. Arrows indicate areas of left ventricular hypoattenuation corresponding to myocardial infarction. (**A**) axial, (**B**) coronal, and (**C**) sagittal plane CT images of the infarct.

**Fig. (2):**
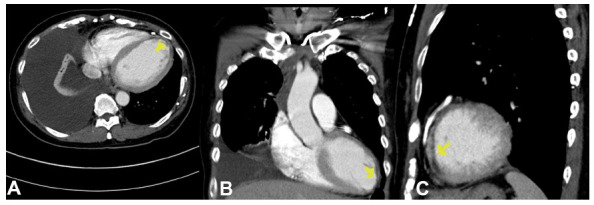
Left Ventricular Myocardial Infarction Detected on Routine Non-Gated Thoracic CT. Incidental myocardial infarction in a patient with lymphoma who underwent thoracic CT for assessing the response to chemotherapy. Arrows indicate areas of left ventricular hypoattenuation corresponding to myocardial infarction. (**A**) axial, (**B**) coronal, and (**C**) sagittal plane CT images of the infarct.

**Table 1:** Indications of non-gated thoracic computed tomographic scans (N=1157).

Staging and follow-up of cancer	577 (49.8%)
Evaluation of solitary pulmonary nodules	175 (15.1%)
Dyspnea and respiratory failure	68 (5.9%)
Abnormal chest radiograph	65 (5.6%)
Cardiothoracic anatomy evaluation	56 (4.8%)
Evaluation for thromboembolism	53 (4.6%)
Cough	35 (3.0%)
Infection	28(2.4%)
Chest wall pain	27(2.3%)
Obstructive lung diseases	27 (2.3%)
Lung cancer screening	24(2.1%)
Suspected or known interstitial lung disease	20(1.7%)
Other (dystonia, trauma)	2 (0.2%)

**Table 2:** Clinical characteristics of individuals at baseline in incidental myocardial infarct negative versus positive group on routine thoracic CT.

-	-	Negative Group	Positive Group	All	p- value
Age, mean (SD), years	-	71.1±13.3	71.4±14.1	71.2±13.3	0.89
BMI*, mean (SD), (kg/m^2^)	-	25.1±4.8	27.1±9.9	25.6±6.3	0.99
Sex (%)	Female	16 (44)	6 (50)	22 (46)	0.75
-	Male	20 (56)	6 (50)	26 (54)	-
	-	-	-	-	-
Race/ethnicity, No. (%)	White	28 (80)	7 (47)	35 (70)	0.26
-	Asian	3 (9)	2 (13)	5 (10)	-
-	African American	3 (9)	0 (0)	3 (6)	-
-	Other	2 (6)	3 (20)	5 (10)	-
	-	-	-	-	-
Hypertension (%)	No	15 (42)	0 (0)	15 (31)	0.009
-	Yes	21 (58)	12 (100)	33 (69)	-
	-	-	-	-	-
Hyperlipidemia (%)	No	13 (36)	1 (8)	14 (29)	0.08
-	Yes	23 (64)	11 (92)	34 (71)	-
	-	-	-	-	-
Diabetes (%)	No	33 (92)	6 (50)	39 (81)	0.004
-	Yes	3 (8)	6 (50)	9 (19)	-
	-	-	-	-	-
CAD** (%)	No	27 (75)	2 (17)	29 (60)	0.0006
-	Yes	9 (25)	10 (83)	19 (40)	-
	-	-	-	-	-
Smoking (%)	No	14 (40)	0 (0)	14 (31)	0.029
-	Yes	21 (60)	10 (100)	31 (69)	-
-	(current/former)	(3/18)	(0/10)	(3/28)	-

Notes:*: Body mass index. **: Coronary Artery Disease.

**Table 3:** Imaging characteristics of the incidental CT infarct positive group.

AHA* Segment(s) Involved by Infarct	Frequency (%)
2,4,5,9,17	2(4.4)
12,16	4(8.8)
1,3,15	1(2.2)
11,14	3(6.6)
13	6(13.3)
8	5(11.1)
7	7(15.5)
Transmurality of infarct	-
>50	3(25)
25-50%	6(50)
<25%	3(25)

Note:*: American Heart Association.

**Table 4:** Coronary artery calcifications in incidental CT infarct negative and positive groups.

-	-	Negative Group	Positive Group	All	p-value
Coronary Calcifications	No	12 (33)	0 (0)	12 (25)	0.023
-	Yes	24 (67)	12 (100)	36 (75)	-
-	Mild	19 (79)	4 (33)	23 (64)	-
-	Moderate	4 (17)	7 (58)	11 (31)	0.011
-	Severe	1 (4)	1 (8)	2 (6)	-

**Table 5:** Outcomes and follow-up in positive and negative CT infarct patient groups.

-	-	Negative Group	Positive Group	All	p-value
MACE endpoint	No	26 (72)	1 (8)	27 (56)	0.0001
-	Yes	10 (28)	11 (92)	21 (44)	-
-	TIA	2 (20)	0 (0)	2 (10)	-
-	Stroke	3 (30)	2 (18)	5 (24)	-
-	MI/CABG	1 (10)	2 (18)	3 (14)	-
-	MI	3 (30)	6 (55)	9 (43)	-
-	CABG	1 (10)	1 (9)	2 (10)	-
	-	-	-	-	-
Number of MACE events	0.3±0.5		1.1±0.5	0.5±0.6	<0.001
	-	-	-	-	-
Timing of cardiac event	After	0 (0)	2 (22)	2 (13)	0.48
-	Before	7 (100)	7 (78)	14 (88)	-
	-	-	-	-	-
Timing of cerebrovascular event	After	0 (0)	1 (50)	1 (14)	0.29
-	Before	5 (100)	1 (50)	6 (86)	-
	-	-	-	-	-
Deceased	No	21 (58)	6 (50)	27 (56)	0.74
-	Yes	15 (42)	6 (50)	21 (44)	-
-	Cancer	12 (80)	3 (50)	15 (75)	0.13
-	Non-cancer	2 (13)	3 (50)	5 (25)	-

## Data Availability

The dataset utilized in this study will be available upon request from the corresponding author [M.M].

## LIST OF ABBREVIATIONS

CT: Computed tomographic
MACE: Major adverse cardiovascular events
MI: Myocardial infarction
PCI: Percutaneous coronary intervention

## STANDARDS OF REPORTING

STROBE guidelines were followed.

## References

[1] Stolz D., Mkorombindo T., Schumann D.M., Agusti A., Ash S.Y., Bafadhel M., Bai C., Chalmers J.D., Criner G.J., Dharmage S.C., Franssen F.M.E., Frey U., Han M., Hansel N.N., Hawkins N.M., Kalhan R., Konigshoff M., Ko F.W., Parekh T.M., Powell P., Rutten-van Mölken M., Simpson J., Sin D.D., Song Y., Suki B., Troosters T., Washko G.R., Welte T., Dransfield M.T. (2022). Towards the elimination of chronic obstructive pulmonary disease: A Lancet Commission.. Lancet.

[2] Kanza R.E., Ayoub S., Bonenfant F. (2020). Incidental, non-gated thoracic CT angiographic detection of proximal right coronary artery total occlusion associated with acute myocardial infarction.. Eur. J. Radiol. Open.

[3] Luciano A., Luigi S., Mancuso L., Vito D.O., De Stasio V., Pugliese L., Donna C.D., Garaci F., Floris R., Chiocchi M. (2023). Incidental findings of acute myocardial infarction detected during ECG-gated and nongated thoracic CTA: A report of four cases.. Radiol. Case Rep..

[4] Kuetting D., Müller A., Feisst A., Luetkens J., Dabir D., Schild H.H., Thomas D. (2018). Incidental cardiac findings in non–electrocardiogram-gated thoracic computed tomography of intensive care unit patients: Assessment of prevalence and underreporting.. J. Thorac. Imaging.

[5] Sverzellati N., Arcadi T., Salvolini L., Dore R., Zompatori M., Mereu M., Battista G., Martella I., Toni F., Cardinale L., Maffei E., Maggi F., Cademartiri F., Pirronti T. (2016). Under-reporting of cardiovascular findings on chest CT.. Radiol. Med. (Torino).

[6] Turkbey E.B., Nacif M.S., Guo M., McClelland R.L., Teixeira P.B.R.P., Bild D.E., Barr R.G., Shea S., Post W., Burke G., Budoff M.J., Folsom A.R., Liu C.Y., Lima J.A., Bluemke D.A. (2015). Prevalence and correlates of myocardial scar in a US cohort.. JAMA.

[7] Cerqueira M.D., Weissman N.J., Dilsizian V., Jacobs A.K., Kaul S., Laskey W.K., Pennell D.J., Rumberger J.A., Ryan T., Verani M.S., American Heart Association Writing Group on Myocardial Segmentation and Registration for Cardiac Imaging (2002). Standardized myocardial segmentation and nomenclature for tomographic imaging of the heart. A statement for healthcare professionals from the Cardiac Imaging Committee of the Council on Clinical Cardiology of the American Heart Association.. Circulation.

[8] Blaha M.J., DeFilippis A.P. (2021). Multi-Ethnic Study of Atherosclerosis (MESA).. J. Am. Coll. Cardiol..

[9] Wetscherek M.T.A., McNaughton E., Majcher V., Wetscherek A., Sadler T.J., Alsinbili A., Teh W.H., Moore S.D., Patel N., Smith W.P.W., Krishnan U. (2023). Incidental coronary artery calcification on non-gated CT thorax correlates with risk of cardiovascular events and death.. Eur. Radiol..

[10] Yacoub B., Kabakus I.M., Schoepf U.J., Giovagnoli V.M., Fischer A.M., Wichmann J.L., Martinez J.D., Sharma P., Rapaka S., Sahbaee P., Hoelzer P., Burt J.R., Varga-Szemes A., Emrich T. (2022). Performance of an artificial intelligence-based platform against clinical radiology reports for the evaluation of noncontrast chest CT.. Acad. Radiol..

[11] Pickhardt P.J. (2022). Value-added opportunistic CT screening: State of the art.. Radiology.

